# Overwhelming Evidence for a Major Role for Herpes Simplex Virus Type 1 (HSV1) in Alzheimer’s Disease (AD); Underwhelming Evidence against

**DOI:** 10.3390/vaccines9060679

**Published:** 2021-06-21

**Authors:** Ruth F. Itzhaki

**Affiliations:** Institute of Population Ageing, University of Oxford, 66 Banbury Road, Oxford OX2 6PR, UK; ruth.itzhaki@manchester.ac.uk; Tel.: +44-(0)1865-250853

**Keywords:** Alzheimer’s disease, herpes simplex virus type 1 (HSV1), apolipoprotein E (APOE), brain, antivirals

## Abstract

This review describes investigations of specific topics that lie within the general subject of HSV1’s role in AD/dementia, published in the last couple of years. They include studies on the following: relationship of HSV1 to AD using neural stem cells; the apparent protective effects of treatment of HSV1 infection or of VZV infection with antivirals *prior* to the onset of dementia; the putative involvement of VZV in AD/dementia; the possible role of human herpes virus 6 (HHV6) in AD; the seemingly reduced risk of dementia after vaccination with diverse types of vaccine, and the association shown in some vaccine studies with reduced frequency of HSV1 reactivation; anti-HSV serum antibodies supporting the linkage of HSV1 in brain with AD in APOE-ε4 carriers, and the association between APOE and cognition, and association of APOE and infection with AD/dementia. The conclusions are that there is now overwhelming evidence for HSV1’s role—probably causal—in AD, when it is present in brain of APOE-ε4 carriers, and that further investigations should be made on possible prevention of the disease by vaccination, or by prolonged antiviral treatment of HSV1 infection in APOE-ε4 carriers, before disease onset.

## 1. Introduction

In 2017, I emphasised the steady increase in the number of publications supporting directly or indirectly the involvement of herpes simplex virus type 1 (HSV1) in Alzheimer’s disease (AD) [1]. Since then, the number has increased further and so greatly that it is not possible in a review of reasonable and digestible length to discuss all or even many of the more recently published papers. In an attempt to manage the super-abundance of evidence for HSV1’s role, I have described only those studies that concern specific features of AD or specific relevant factors; of course, that precludes mention of the most original papers as, by definition, they could not be categorised. I must, therefore, apologise for omitting a number of interesting and important studies.

To reiterate the concept of a role for HSV1 in AD, much evidence indicates that HSV1 can enter the brain, probably in middle age, and can reside there in latent form. Stress, inflammation, and other events can lead to reactivation of the virus, causing a productive infection and consequent damage which, it is suggested, is likely to be greater in people who carry the type 4 allele of the apolipoprotein E gene (APOE-ε4). Recurrent reactivation causes cumulative damage, leading eventually to AD. The conclusion was that HSV1 in brain, in combination with carriage of an APOE-ε4 allele, confers a high risk of developing AD. HSV1 alone, i.e., HSV1 in brain of non-APOE-ε4 carriers, confers a much lesser or no risk. Our finding that in the peripheral nervous system, APOE-ε4 is a major risk factor for cold sores (herpes labialis) provided strong indirect support for this conclusion [2].

Some articles which appraise the concept appear to have certain misunderstandings or misconceptions, so it seems necessary to refute them yet again: firstly, there is often lack of awareness that “controls” can be infected but asymptomatic, i.e., that *infected* does not necessarily mean *affected*, so that broadly similar values of frequency/prevalence of a microbial infection in controls and diseased subjects do *not* preclude a role for that microbe in the disease. Host genes or other factors might well determine response of an individual to a microbe or agent. For example, at least 80% of people are infected with HSV1 but only about one fifth of them suffer from cold sores: the other four fifths, being asymptomatic, would be listed as controls. Secondly, it is sometimes stated that AD patients are more susceptible to infection with HSV1 than controls - but this is vitiated by the fact that the prevalence of HSV1 in brain is not much lower in controls than in AD patients [2]. Thirdly, the idea that APOE-ε4 carriers are more likely to be infected with HSV1 is demonstrably incorrect: 80+% of most populations are infected with the virus but only some 25–30% are APOE-ε4 carriers.

## 2. Studies Relating HSV1 to AD Using Stem Cells

Several very useful studies have been carried out using differentiated stem cells. Aiuto et al. [3] used HSV1-infected 2D and 3D cultures of neuron-like human induced pluripotent stem cells (hipsc) derived from skin fibroblasts as a model for HSV1–human–CNS interactions. The authors found that the cells were permissive for HSV1 infection, that a quiescent state resembling HSV1 in animal models could be established, that in the 3D cultures the virus travelled from the periphery to the centre of the structure, and that reactivation from the quiescent state could be achieved. Reactivation was less efficient in the 3D cultures compared with the 2D cultures, thereby resembling the lesser frequency of reactivation in the CNS compared to the PNS, in lab animals infected with HSV1. Reactivation caused neurodegeneration of neuronal processes and cell-cell fusion, which led to the formation of neuronal syncytia.

In a later study, the same group [4] found different patterns of beta amyloid 42 (Aβ42) accumulation in HSV-1 infected 2D and 3D neuronal cultures. The 2D neuronal cultures showed Aβ42 almost always in HSV1-infected cells (detected by staining the major HSV1 transcription factor, immediate early ICP4 (infected cell polypeptide 4, a marker of HSV1 replication) or in infected cells exposed to antivirals, whereas 3D brain organoids showed Aβ42 mainly in non-infected cells surrounding HSV1-infected cells. The authors suggest that because brain organoids better evoke the features of a developing brain than 2D cultures, they provide a more suitable model for investigating the involvement of HSV1 in AD pathology.

**Figure 1:**
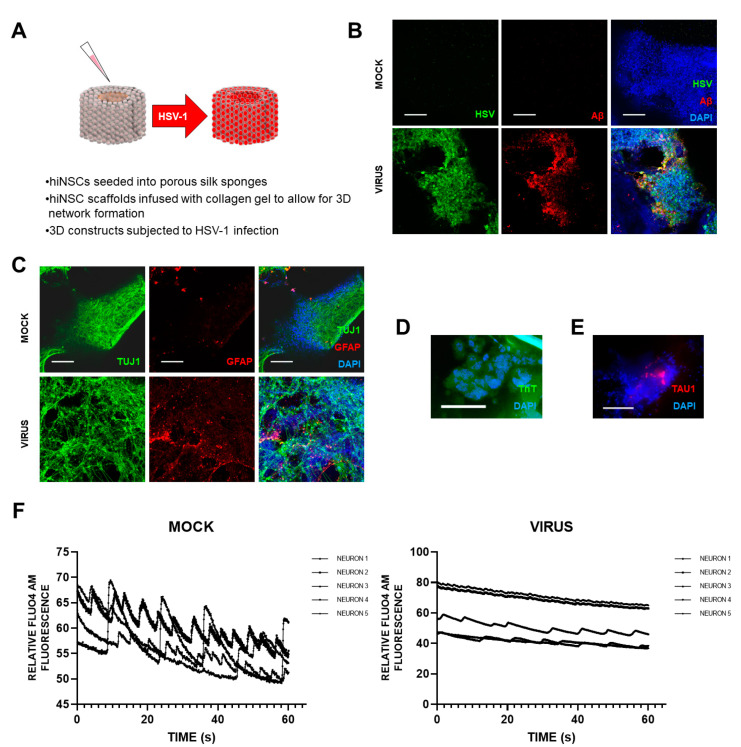
Effects of HSV1 infection of 3D brain-like models, showing changes similar to those in AD brain. Scaffolds were seeded with human-induced neural stem cells (hiNSCs), of APOE-ε3/4 genotype, and allowed to mature for 4 weeks. They were then infected with HSV1 for I week, after which, samples were harvested for multiple assays. (**A**) Schematic of 3D herpes-induced brain-like tissue model of AD. (**B**) Fluorescent immunostaining mages of 3D donuts showing herpes simplex virus 1 (HSV) and beta amyloid (Aβ) expression in virus-infected samples. (**C**) Fluorescent immunostaining images of 3D donuts showing pan-neuronal marker beta III tubulin (TUJ1) and glial fibrillary acidic protein (GFAP) expression in virus-infected samples. (**D**) Thioflavin T (ThT) histological staining showed the presence of Aβ fibrils in infected cells. (**E**) Fluorescent Tau immunostaining showing expression within plaque-like formations (PLFs) present in HSV-1-infected samples. (**F**) Live Fluo4AM calcium imaging showing representative traces of neuronal firing within mock or virus-infected hiNSC scaffolds. All scale bars are 100 μm.

Cairns et al. [5] recently set up a novel and exciting 3D bioengineered brain model using human-induced neural stem cells (hiNSCs), of APOE-ε3/4 genotype (D. Cairns and D. Kaplan, personal commun.), to examine the effects of HSV1 infection relating to AD. These stem cells spontaneously differentiate into multiple neuronal and glial subtypes and do so rapidly—after only about 4 days. The authors stated that the system allows visualization and quantification of neurite patterning and networks and electrophysiological readouts in real time They found that on infection, the model mimics human disease, with multicellular amyloid plaque-like formations, gliosis, neuroinflammation, and decreased functionality (Figure 1) in that there was significantly less electrophysiological activity, comparable to the impaired functionality of AD patients. The authors stressed that these AD-like changes occurred in the absence of any exogenous mediators that might regulate or induce AD. Further, addition of the antiviral valacyclovir (VCV) reduced the magnitude of the changes, relating more directly to antiviral effects on infected brain than did previous studies using Vero cells. Their AD model strongly supports a causal role for HSV1 in brain (with APOE-ε4) in AD and supports also the earlier studies on antivirals by the present author’s laboratory ([6] et seq.).

It is worth noting that Choi et al. [7], who used 3D cultures of familial AD (FAD) patient-derived ipsc, or genetically modified human stem cell lines with APP or APP/presenilin mutations, i.e., neuronal models for FAD, mainly relating to Aβ production, stated that neurons derived from sporadic AD patients gave variable results in Aβ levels, showing increased levels in only a few cases.

## 3. Usage of GV971 to Treat AD and the Case for Using a Related Compound, Fucoidan

The study by Cairns et al. [5] strongly supports the use of antiviral treatment for AD patients, and a clinical trial of VCV to treat AD patients is ongoing at Columbia university. Another clinical trial treating AD patients, described by Cummings et al. [8], is yielding encouraging results: a phase 2 trial of GV971, a compound derived from brown algae which consists of polysaccharides—linear sodium oligomannate molecules—of a range of sizes. This, they suggest, may reduce brain inflammation as well as systemic inflammation through effects on the gut microbiome, and very recently, the positive results of a phase 3 trial of GV971 have been reported by Xiao et al. [9]. The latter authors stated that GV971 demonstrated significant efficacy in improving cognition, with sustained improvement across all observation periods of a 36-week trial, and it was safe and well-tolerated. However, an alternative—or perhaps additional—mechanism might operate via the drug acting as an antiviral agent against HSV1 [10]. Marine-derived polysaccharides have been shown to have a variety of bioactivities, including antiviral effects [11], and anti-bacterial effects.

The antiviral activities of the marine-derived polysaccharides are usually related to the specific sugar structure, molecular weights and their degree of sulfation. (On enquiry, the present author was told by the Company, Green Valley, that GV971 was not sulphated, so she recommended that they assess a sulphated version for possible future usage as treatment for AD.) Fucoidans (known also as fucans), a group of polysaccharides derived also from brown algae, are sulphated fucans; they have long been used as food supplements because of their apparent protective action against various illnesses. They have both antiviral and virucidal activity against HSV1 [12], their mechanism of action being attributed to their inhibiting the initial attachment of the virus to the host cells. The inhibition is probably mediated by interactions of the fucoidans with positively charged domains of the viral envelope glycoproteins, which direct the attachment of the virus to heparan sulphate proteoglycans on the host cells’ surface.

Sulphated fucans were found to be very effective in reducing the levels of Aβ and especially AD-like tau (P-tau) produced in cultured cells infected with HSV1, the activity of the fucoidan derived from *Undaria pinnatifida* against the HSV1-induced production of Aβ and P-tau being particularly effective [13]. Further, when the fucoidan was added to infected cells together with the most commonly used anti-HSV antiviral agent, acyclovir (ACV), which interferes specifically with viral DNA replication, the combined effect was synergistic—appreciably more effective than either agent alone in decreasing levels of P-tau and Aβ. Presumably, fucoidan reduced the number of viruses entering the cells and, in the case of those viruses that did manage to enter, ACV then inhibited the replication of their DNA

As to fucoidan’s usage, an in vitro study suggests that it might well combat SARS-CoV-2 also: Kwon et al. [14] investigated the relevant antiviral activity of fucoidan and other highly sulphated polysaccharides, as well as heparin, heparan sulphates, and other glycosaminoglycans(GAGs), based on their finding that heparin has exceptional binding affinity to the spike protein (S-protein) of SARS-CoV-2 They used Vero cells, which express ACE2, the SARS-CoV-2 virus receptor, and measured the binding of the above compounds to the SARS spike protein CoV-2 S-protein by surface plasmon resonance. Fucoidans extracted from the seaweed Saccharina japonica and chemo-enzymatically synthesized derivatives of heparin as well as heparin itself were found to have significant antiviral activity. The authors, therefore, suggested that as SARS-CoV-2 infects a wide range of tissues that have adequate ACE2 levels, including the nose and the gastrointestinal tract, potential delivery of these substances, including the fucoidans, could be through a nasal spray, metered dose inhaler, or oral delivery. They added that the fucoidans, when taken orally, are considered “Generally Recognized as Safe”.

The next section deals with links between COVID-19. HSV1, and APOE.

## 4. COVID-19 Effects on Herpesvirus Reactivation, and Dependence of COVID-19 Severity on APOE

These seemingly disparate topics are, in fact, connected, as described in the articles that follow. Three very recent studies have shown that severe COVID-19 causes reactivation of herpesviruses, consistent with the many previous studies showing that peripheral infections, by causing inflammation, can reactivate HSV in brain. Le Balc’h et al. [15] reviewed all virology results for patients admitted to Rennes University Hospital for COVID-19-associated severe acute respiratory distress syndrome (ARDS) between 3 March 2020 and 15 April 2020. SARS-CoV-2 infection was confirmed by PCR. Patients mechanically ventilated for longer than 7 days and who had negative PCR for HSV and cytomegalovirus (CMV) were included in the analysis. HSV and CMV replication were measured by quantitative real-time PCR on tracheal aspirates twice a week for each patient. Herpesviridae reactivation was defined as two consecutive positive HSV or CMV PCRs on tracheal aspirates. Of the total of 38 severely affected COVID-19 patients, mean age, 59 years, 18 (47%) presented at least one viral pulmonary reactivation; of these, nine had HSV reactivation alone, two had CMV reactivation alone, and seven had co-reactivation. (As some of the patients were herpesvirus-negative their aspirates could not have shown reactivation).

Xu et al. [16] performed serial follow-up tests of pathogen next-generation sequence (NGS) on a severely ill patient aged 73, including tests on blood, sputum and bronchoalveolar lavage fluid (BALF). The blood pathogen NGS report revealed the onset of both VZV and HSV1 reactivation.

Simonnet et al. [17] investigated all patients admitted for SARS-Cov-2 infection between 16th March and 6th August 2020, for EBV DNA, CMV DNA and HHV-6. Nasopharyngeal and throat swab samples were obtained at admission from all patients, who were tested using real-time RT PCR assays to identify SARS-CoV-2 infection. Blood EBV DNA, CMV DNA and occasionally HHV-6 DNA were routinely quantified for patients considered at high risk of reactivation, until ICU discharge or death. Five patients in the cohort (15%) presented no viral DNAemia detection. Twenty patients (59%) were positive for one virus (EBV in 19 cases and HHV-6 in one case), 7/34 patients (20%) were positive for 2 viruses (EBV and CMV in three cases, EBV and HHV-6 in four cases) and 2/34 patients (6%) were positive for the three viruses. Thus, viremia with EBV, CMV, and HHV-6 was detected in 28 (82%), five (15%), and seven (22%) patients, respectively. EBV reactivation occurred early after ICU admission and was associated with longer ICU length-of-stay.

Heneka et al. [18] have commented on the possible long-term effects of COVID-19, describing the neurological deficits of infection with Sars-CoV-2 in many patients. One third of COVID-19 patients on discharge show cognitive impairment and motor deficits, and the authors stress the relevance of the fact that COVID-19 is most severe in the elderly. Severely affected COVID-19 cases experience high levels of proinflammatory cytokines and acute respiratory dysfunction and often require assisted ventilation—all factors that might cause cognitive decline, either as a direct consequence, or from immune dysregulation, or via enhancement of pre-existing cognitive deficits. They conclude that those surviving COVID-19 are at high risk of subsequent development of neurological disease and particularly of AD. This conclusion is somewhat surprising, as there is no firm evidence yet of SARS-CoV-2 presence in brain and, therefore, arguments *against* its having a role in AD would, presumably, be much stronger than those often raised against the putative role in AD of HSV1 (the presence of which, in contrast, in brain of many elderly people is firmly established). If, instead, the effect of SARS-CoV-2 is postulated to be indirect, via inflammation, this could act as a reactivator of HSV1 in brain which, in APOE-ε4 carriers would lead to AD, i.e., would not be attributable to SARS-CoV.

There is also an APOE-COVID-19 connection, based on the fact that APOE-ε4 is a risk for dementia and for delirium, and that in older adults, pre-existing dementia and delirium are risk factors for severity of COVID-19. Kuo et al. [19] investigated a possible direct link between APOE-ε4 and severity of COVID-19. They found that APOE-ε4 homozygotes were indeed at increased risk of severe COVID-19 infection and that the association was similar after removing participants with *APOE-* ε4-associated diseases that were linked also to COVID-19 severity. The present author had planned to seek a possible APOE-SARS-CoV2 link, because studies by her lab had revealed that APOE genotype determines susceptibility to a pathogen, or severity of damage by the pathogen, in a number of infectious diseases, including herpes labialis [2]. The diseases were selected on the basis that the cell surface receptors for entry of the pathogen into cells were receptors also for the protein apoE (coded for by the APOE gene), and that the different isoforms of the protein (types 2,3,4) might compete to a different extent with the pathogen for cell entry [20].

An explanation for the results of Kuo et al. is that the heparan sulphate proteoglycan (HSPG)-receptor- binding domain of apoE has broad antiviral activity, directly blocking viral entry for HSV1, HSV2 and HIV [21,22]. SARS-CoV2 also initially attaches to the same non-specific receptors, HSPGs, as does apoE (although it then binds to its main receptor, the angiotensin converting enzyme-2, ACE2), and so the APOE genotype effect might result from apoE competing for HSPG binding. In fact, a related coronavirus—SARS-CoV—has previously been shown to be inhibited by lactoferrin through competition for HSPG binding. The extent of the possible apoE-SARS-CoV-2 competition might differ among the apoE isoforms (the mutations producing these isoforms flank the apoE HSPG-receptor binding domain). Alternatively, SARS-CoV2 systemic damage causes inflammation in brain that might reactivate latent HSV1 resident there; indeed, as Helms et al. [23] have found, neuropsychiatric symptoms do not require invasion of the CNS by SARS-CoV2. In addition, a recent study by Thakur et al. [24] on COVID-19 neuropathology states that “Our findings indicate that the levels of detectable virus in COVID19 brains are very low and do not correlate with the histopathological alterations”.

## 5. Effect of Treatment with Antivirals for HSV and VZV Infection *prior* to Onset of Dementia

Some very surprising but potentially important results, if verified, dealing with the consequence of herpesvirus reactivation have been reported. To put them into context, the results of three relevant studies previously described [25] are well worth repeating: Chen et al. [26] compared herpes zoster (HZ—shingles) patients with controls and found there was a slightly increased risk associated with HZ (HR (Hazard ratio) 1.11; 95% CI, 1.04-1.17). In addition, they compared HZ patients treated with antivirals (AVT), all of which target VZV as well as HSV1, with untreated HZ patients, and found a greatly reduced risk of subsequent dementia in the following 6.2 years (HR = 0.55, 95% CI, 0.40–0.7). Tzeng et al. [27] identified subjects aged ≥ 50 years during the year 2000 who were newly diagnosed with HSV (HSV1 or HSV2) infection, with very probably severe, recurrent symptoms of herpes labialis and/or genital ulceration. They were compared with a control group of 25,086 people with no HSV infection during the index year. The risk of dementia in the HSV-infected group was very much higher (HR 2.564, 95% CI, 2.351–2.795; *p* < 0.001). Most of the infections were caused by HSV1, although HSV2 conferred a small risk. AD and vascular dementia showed similar risk profiles. Anti-herpetic treatment dramatically reduced later incidence of dementia in the 10-year follow-up (to 2010) period (HR, 0.092, 95% CI, 0.079–0.108, *p* < 0.001); protection was greater in those treated for longer time periods (>30 days versus <30 days).

More recently, a study by Bae et al. [28] using National Health Insurance Service data in Korea analysed 229,594 individuals aged ≥ 50 years. Patients with HZ had a higher risk of dementia (adjusted HR, 1.12, 95% CI, 1.05–1.19). Of the 34,505 patients with HZ, 28,873 (84%) had received antiviral treatment (acyclovir, famciclovir, or valaciclovir). The treated group showed a significantly lower risk of dementia (HR 0.76, 95% CI, 0.65–0.90). No details of duration or dosage were available in the databank. However, these results broadly resemble those of Chen et al, and Tsai et al.

Schnier et al. [29] investigated the linked electronic health records of 2.5 million individuals aged ≥ 65 years in national observational cohort studies on HSV infection in populations in four different countries. The criterion for diagnosis of HSV infection was not stated in the records, so it is uncertain whether it was based on serology or on appearance of herpes labialis, or of genital ulcers (usually caused by HSV2). The authors compared infected subjects who had not been treated with antivirals, so presumably, their symptoms were mild, with subjects not examined for HSV infection, though most would very probably have been HSV1-infected (as is about 80% of the population). Unsurprisingly, the former group showed only a slight increase in risk of AD/dementia (HR 1.18). The much greater risk—HR 2.56—found by Tzeng et al. was presumably, attributable to the latter authors comparing subjects with presumed severe HSV1 symptoms—and therefore very probably APOE-e ε4 carriers. As for the effect of antiviral treatment, Schnier et al. examined HSV1-infected subjects with (presumed, as mentioned above)) mild infection, comparing antiviral-treated (single prescriptions for 1–2 weeks) with those not antiviral-treated. They found that in the Danish and Welsh cohorts, people who were prescribed at least one dose of herpes antiviral medication (mainly ACV and VCV) had up to only 11% percent decrease in dementia risk, which the authors considered might result from confounding factors, while the two other cohorts, in Germany and Scotland, showed no altered risk. The dementia outcome was unaffected by type of herpesvirus. Again, these data were in strong contrast to those of Tzeng et al. who found that AVT-treated patients had a very greatly reduced risk of dementia, quite possibly reflecting the longer duration of treatment.

A very recent study by Lopatko Lindman et al. [30] in Sweden investigated 265,172 subjects aged ≥ 50 years, diagnosed with VZV or HSV1 infection, between the years 2005 and 2017, some of whom were treated with antivirals, mainly VCV and ACV. Data of viral diagnoses and antiviral treatment were collected from two nationwide databases: the National Patient Register (NPR) and the Swedish Prescribed Drug Register. The authors stated that HSV and VZV diagnoses in Sweden are usually based on physical examinations for the presence of typical rashes and their location; presumably, this means that in the case of HSV1 the subjects had severe herpes labialis and in the case of VZV, the subjects had shingles. Controls without herpes diagnoses and without antiviral treatment were matched randomly in a 1:1 ratio by sex and year of birth. There were more cases of VZV infection than of HSV infection (4323 and 5722 with no antiviral treatment, respectively, and 24,045 and 6510 with antiviral treatment, respectively, and in some cases the subjects revealed infection with both viruses). The authors compared subjects without antiviral treatment to those prescribed antiviral drugs at least once. They were unable to study any dosage- or duration-related effects, as there was no adequate information in the databases. However, the standard dosage for treatment of herpes zoster reactivations, according to Swedish clinical guidelines, is VCV 1000 mg three times a day for 7 days or ACV 800 mg five times a day for 7 days. They found that VZV and/or HSV-infected subjects not treated with antivirals had an increased risk of dementia (adjusted HR 1.50, 95% CI, 1.29 to 1.74), and that antiviral treatment reduced the risk of dementia (adjusted HR 0.89, 95% CI, 0.86 to 0.92).

A major feature which was not considered in the above studies, as no APOE genotypes were provided in any of the above databases, is the effect of APOE-ε4. There is good evidence that this allele, in combination with HSV1 infection in brain, is a risk for AD. Thus, those HSV1-infected subjects who are APOE-ε4 carriers would be at greater risk of AD/dementia than the HSV1-infected group as a whole who, in contrast, would show little or no risk because the proportion of APOE-ε4 carriers is fairly low anyway and decreases with old age. As APOE-ε4 is a major risk for herpes labialis (accounting for about 65% of cases [2]), cold sore sufferers are presumably at high risk of AD.

The involvement of VZV in the study by Lopatko Lindman et al. supports the four previous findings [26,28,29,31] in showing that VZV, like HSV1, confers a risk of dementia (supported further by epidemiological and serological research on VZV by the present author and her colleagues [32,33].

It is worth mentioning that epidemiological studies on VZV are relatively straight-forward, as shingles is an easily diagnosed illness, whereas with HSV1, cold sores if mild are often not reported to a physician, let alone diagnosed; also, they can vary in frequency, severity and duration and quite often are treated only by the sufferer. Such details would not be recorded in a databank, so cold sores are scarcely suitable as markers of HSV1 infection for epidemiological studies (unlike shingles—good makers for VZV), especially as they affect only some 25–40% of HSV1-infected people; the remaining 60–75% are asymptomatic even though infected. Importantly, studies on cold sores have the further disadvantage that it is not known whether they indicate reactivations of latent HSV1 in the brain as well as of latent HSV1 in the periphery; a similar uncertainty exists also about whether serum antibodies, which indicate the presence of HSV1 antigens in the periphery, indicate also activity of HSV1 in the brain.

As to whether or not the action of VZV is direct, i.e., resulting from its being present in brain and its subsequent reactivation there, or whether instead it is indirect, acting via inflammatory processes which cause reactivation of HSV1 resident in brain is uncertain. Such indirect, inflammation-induced reactivation of HSV1 in the CNS could presumably be caused also by infective agents other than VZV in the PNS [34], including as mentioned before, SARS-CoV-2. In fact, the present author’s lab sought VZV DNA in AD and aged control brains [35] but did not detect it, whereas Hemling et al. [36] did find it, though they did not detect HSV1 DNA (surprisingly, in the case of VZV, as the sensitivity of their detection of the DNA of both viruses was far lower than that of Lin et al.). Involvement of VZV in dementia has been suggested by other approaches, such as the study by Grahn et al. [37] which showed that after acute VZV infection, patients who had suffered predominantly CNS manifestations showed significant cognitive decline 3 years later.

The results of Schnier et al. showing little or no effect of antiviral treatment on subsequent dementia differ from those of the two Taiwan studies, and those of Bae et al. and Lopatkin Lindman, all of which showed that pre-treatment of herpes infections with antivirals reduced the risk of subsequent dementia. This very probably relates to the much longer duration and/or more frequent usage of antiviral treatment, in the studies in which duration data were available, than in that of Schnier et al.

The actual sequence of events affording protection against dementia by prior antiviral treatment is unknown. As the present author and Richard Lathe pointed out [38], the protective effect is extremely puzzling, because after each treatment the antiviral would have remained in the body for only a few days, and much would have been excreted. We suggested one possible explanation, based on research by my group some 24 years ago [2]: that the virus, which resides in the PNS of most adults, might travel from the PNS to CNS in late middle age as the immune system declines. Assuming that this suggestion is correct, we speculated that antiviral treatment might prevent the transit of HSV1 into the brain, or perhaps delay its transit. If so, extending the Taiwan survey for 5–10 years could have determined whether dementia cases later increased after 2010 in the treated cohort [38]. Investigations to seek HSV1 DNA *post mortem* in the brain of any such subsequent cases of dementia, and of those who remained free of the disease, might have helped to elucidate the situation.

Alternatively, in older people, if the virus has already reached the brain, the antiviral might reduce the frequency of reactivation—as intimated by Lopatko Lindman et al. However, an argument against both suggestions would be that even the longer durations of treatment or the more frequently given doses, would presumably have been rendered ineffectual eventually by subsequent passage of the virus to the CNS, or resumption of its usual reactivations in the CNS. Thus, the mystery of the effectiveness of prior antiherpetic treatment in reducing the risk of subsequent dementia remains to be solved.

It would be of great interest to discover whether or not VZV does reside in the brain and to discover whether HSV1 is the sole neurotropic virus (or bacterium) resident there. Other more minor points of interest which need to be elucidated, though they are often not specified in such databases, is whether HSV means HSV1 or, far less likely, HSV2 - genital herpes; also, APOE genotypes, and details of the type, duration, etc., of the antiviral used would be valuable.

## 6. Involvement of VZV in AD/Dementia

Apart from in the studies described in the preceding section, VZV has not so far been suggested as a prospective cause of dementia. A possible argument against its involvement is the fact that it usually reactivates only once, rarely a second time. Recurrent reactivations seem to be a prerequisite for the development of dementia, as progression of the disease occurs over many years. However, as mentioned above, it is unknown whether or not dementia might be caused by VZV if present in brain or via inflammation (a known reactivator of latent HSV1) caused by peripheral VZV infection leading to HSV1 reactivation in brain. In the case of herpes zoster (shingles) sufferers, both Chen et al. [26] and Bae et al. [28] found shingles was associated with a clear but small risk of AD/dementia, and a significantly lower risk in those treated with antivirals.

As to whether an allele of APOE confers a risk of shingles is uncertain: Pirtilla et al. [39] examined the APOE genotypes of 41 patients with HZ and found no differences in allele frequency between the patients and controls. In contrast, my group (in one of several studies on diseases in which the pathogen involved shared cell surface receptors with a specific apoE isoform and thus might compete for cell entry), found that women homozygous for APOE-ε4 had a higher-than-normal risk of shingles [40]. However, both studies had only a small number of participants, so they need to be repeated.

## 7. Possible Involvement of HHV6 in AD

Another herpesvirus which might play a role in AD is HHV6. In AD patients, we found that HHV6 frequency was high (70%), but in age-matched controls, it was low (40%) and there was much overlap with HSV1, 54% of the AD patients’ brains harbouring both viruses. In contrast, HSV1 was detected by the Itzhaki lab in the brain of a high proportion of aged controls (in other words, these controls were infected but were asymptomatic, and so were classed as controls, as occurs in many microbial diseases), as well as of AD patients [41], the salient difference being that most of the AD patients, but very few aged controls, carried an APOE-ε4 allele, so HSV1 in brain and APOE-ε4 together were proposed as a major risk factor for AD; also, it was discovered that APOE-ε4 is a risk for herpes labialis [2]) However, HHV6 was not directly associated in AD patients with APOE-ε4. We suggested that although HHV6 might be an opportunistic infection, alternatively, it might enhance the damage caused by HSV1 and APOE-ε4 in AD [35]; it had been found to do so in the case of certain viruses in cell culture and in animals.

Readhead et al. [42] who analysed the transcriptomes of brain samples from AD patients and controls, using four independent cohorts, detected herpesviruses 6A and 7, as well as HSV1 (unfortunately, the data of Readhead et al. have sometimes been misreported, in stating wrongly that they did not detect HSV1.), in elderly and AD brains, the levels of HHV6 and HHV7 being higher in the AD samples than in the controls, in three of the four cohorts. Analysis of protein and mRNA levels suggested that infection with these viruses caused changes in several transcriptional regulators (including several regulators of amyloid precursor protein-processing and AD risk-associated genes). The data of Readhead et al. [42] argued against HHV6 and HHV7 being merely opportunistic infections, as did those of Lin et al. [35], in that their data revealed associations between levels of those viruses and levels of various characteristic AD features.

However, these results elicited two combative responses: Allnut et al. [43] re-analysed the bioinformatic data from two of the sources investigated by Readhead et al., using the PathSeq tool developed by the BROAD Institute, which screens over 25,000 microbes, including 118 human viruses, and analysed also DNA from brain of AD patients and controls for HHV-6A and HHV-6B by droplet digital PCR (dd PCR). They found that the frequency of detection for any virus was extremely low and was not significantly different between AD (nor between sub-classifications of AD) and non-AD brains. Surprisingly, Allnut et al. did not discuss the apparent absence of HSV1 transcripts. In fact, HSV1 DNA presence in normal elderly brains as well as in AD brains has been well established: data from several hundred studies, using a great variety of methods, are consistent with HSV1 presence in brain, with virus activity and state of latency or reactivation, and many are consistent with the concept that HSV1 in brain of APOE-ε4 carriers confers a high risk of AD. Further, the finding that APOE-ε4 is a risk for herpes labialis in the PNS—caused usually by HSV1—is consistent with the concept that HSV1 in APOE-ε4 carriers is particularly damaging in the nervous system.

The fact that Allnutt et al. concluded from the similarity of their values of frequency/prevalence, or, amount/level/abundance, of a specific virus in ADs and controls, that HHV6 is probably not involved in AD, indicates that the general point made at the start of this article needs to be stressed: that controls too can be infected. In addition, in their ddPCR experiments seeking HHV6 and HHV7 in brain samples, checks for false positives were mentioned, but checks for false negatives were not mentioned—even though their findings were almost wholly negative. Other useful information would have included details of the preparation of the DNA they obtained for the study, because in certain extraction procedures there is a high risk of loss of some DNA species, if present at a very low level.

Chorlton [44], on analysing the supplementary data of Readhead et al. with KrakenUniq, a method which he described as a fast yet highly sensitive method, detected no HHV7, and detected HHV6A in 13% of the samples that had been reported with the highest HHV6A abundance; 13%, as he stated, was consistent with previous research and he commented that almost all previous reports have relied on nucleic acid amplification for detection of HHV6A and HHV7, which is far more sensitive than shotgun sequencing. Further, based on Supplementary Table S2 from Readhead et al., he calculated viral prevalence based on their modified Viromescan, and found that in the main Mount Sinai Brain Bank (MSBB) dataset, HHV6A and HHV7 were detectable in 27% and 30% of 602 brain samples, respectively. Extraordinarily, the prevalence of variola virus (though found in only a single tissue) in the MSBB, was 97.5%—despite the fact that variola virus is the cause of the eradicated disease, smallpox.

These two studies, therefore, do not clarify, let alone answer, the question of whether or not HHV6 is involved in AD, and, in fact, even its prevalence in the brain of AD patients and age-matched controls is still uncertain.

## 8. Vaccination Protection against Risk of AD and of HSV1 Reactivation

Whichever viruses are involved in AD, one would assume that to prevent their action, specific vaccines would be needed against each one. However, vaccines—in particular Bacillus Calmette Guérin (BCG), which was designed to protect against tuberculosis—have been known since the early years of the 20th century to induce diverse non-specific (off-target) effects (see reviews [45,46,47]). These effects include reduction of infant mortality independently of its effect on tuberculosis, e.g., in West Africa a 50% reduction in overall mortality was demonstrated in children vaccinated with BCG—too large an effect to be explained by protection against tuberculosis alone [46]. More recently, these findings have been validated in randomized controlled trials (RCTs) and in a meta-analysis of three RCTs investigating mortality reduction amongst low birth-weight infants [46]. The mortality reduction in infants by BCG was attributed to protection against unrelated infectious agents, particularly against respiratory tract infections, and to protection against neonatal sepsis [48].

Arts et al. found that BCG reduces the viraemia induced in volunteers by experimental attenuated yellow fever vaccination (YFV), and confirmed several previous studies showing that it enhances ex vivo cytokine responses to unrelated pathogens. The authors suggested that epigenetic reprogramming of monocytes is induced by BCG vaccination, accompanied by significantly altered responses of innate immune cells, as shown by the higher pro-inflammatory cytokine production (TNFa, IL-1b, IL-6) of peripheral blood mononuclear cells (PBMCs) from BCG-vaccinated volunteers, compared to placebo-treated individuals. This high cytokine production capacity leads to a rapid local antimicrobial response and subsequent elimination of the pathogen, thereby preventing a systemic reaction and preventing high levels of circulating cytokines. The authors stressed that despite the lower virus load, BCG vaccination did not affect generation of protective anti-yellow fever antibodies and did not affect the specific effect of YFV. This suggests that BCG might improve the antigen-presenting capacity and adaptive responses, consistent with another study that showed the beneficial effects of BCG on the response to subsequent trivalent influenza vaccination [49]. The latter study found also that the trivalent influenza vaccination too exerts nonspecific effects on cytokine responses elicited by various pathogens.

Animal studies have yielded consistent data, such as the finding that immunization with BCG alleviates neuroinflammation and cognitive deficits in APP/PS1 mice (a model for familial AD) via the recruitment of inflammation-resolving monocytes to the brain [50]. The authors showed that BCG treatment reversed the cognitive decline to the extent observed in the control group vaccinated with 4Aβ1-15 (four tandem repeats of GPGPG-linked Aβ1-15 sequences), but did not reduce the Aβ burden in the brain. They suggested that BCG exerts a beneficial immunomodulatory effect in APP/PS1 mice through mitigation of systemic immune suppression, induction of interferon-γ response and alleviation of the neuroinflammatory response.

An important study of off-target effects of vaccines was that of Verreault et al. [51] who found that vaccines for diphtheria, tetanus, poliomyelitis, and influenza were protective against the development of AD. In a comment on this article, it was suggested that the reduction in risk, caused presumably, by reduced infection and hence reduced inflammation, would decrease the frequency of reactivations of HSV1 in brain, thereby decreasing the risk of AD [52]. Consistently with the data of Verreault et al, a later study [53] showed that the BCG vaccine reduced AD incidence in bladder cancer patients; the authors suggested that BCG increases anti-inflammatory cytokines in the brain, and thus reduces neuro-inflammation.

In the reviews of Pittet and Curtis [47] and Adesanya et al. [45], very relevantly to the HSV1-AD concept several studies on the effects of BCG on recurrence of herpes labialis and of genital herpes were analysed. These included Hippman et al. [54] who investigated the efficacy of a single intracutaneous BCG injection in preventing recurrent herpes labialis in Tine-test-negative (i.e., negative for infection with M. tuberculosis) subjects, most of whom had had a recurrence rate at least of one per month for many years. One hundred and nine patients were BCG-vaccinated (the Tine-test then became positive in 106 out of the 109 patients), and their recurrence rates were compared with their rates before vaccination, i.e., each acted as his/her own control. After vaccination, no subjects suffered a recurrence for at least 4–6 months. Hippman et al. followed also a different group of subjects for up to 10 years and showed that the BCG effect decreased over the years, although 21 subjects (19%) had still not had any recurrence after 3 years, and 10 subjects (9%) had had none after more than 6 years. These reductions of frequency and duration of recurrence after BCG were statistically significant (*p* < 0.01). As the latter and all the other studies above showed a marked reduction in recurrence frequency, consistent with the suggestion that vaccination reduces HSV1 (and HSV2) reactivation, Pittet and Curtis recommended further investigation of BCG’s effect on HSV recurrence; these should include, in particular, further properly controlled RCTs, and information on the influence of BCG strain, optimal dosing and need for repeat dosing.

## 9. Anti-HSV Serum Antibodies and Risk of AD

As mentioned earlier, in the section on effect of treatment with antivirals for HSV and VZV infection prior to dementia onset, there have been a number of studies investigating the presence, level, and avidity (relative strength of antibody-binding to antigens) of antibodies to HSV1, specifically immunoglobulin G (IgG) and immunoglobulin M (IgM); the former denotes presence of HSV1 in the periphery, and the latter its reactivation there. Whether these data provide information about HSV1 action only in the PNS is uncertain, but it seems likely that they also apply at least in part to the CNS.

Letenneur et al. [55] surveyed a group of people aged 65 over a 14-year period and found, using serum anti-HSV1 IgM as a marker of recent HSV1 reactivation in the PNS, that those who experienced HSV1 reactivation had an increased risk of developing AD compared with those who were IgM-negative. Subsequently, it was found that high HSV1-IgG antibody titres were more frequent in patients compared to age-matched controls [56]. In addition, a positive correlation was reported between HSV1- specific IgG titres and the cortical volumes of brain regions mainly affected in AD, in patients with mild AD (Mini-Mental State Examination (MMSE) 18–23) [57] it was therefore suggested that HSV1-specific humoral immunity might have a protective role in the early phase of AD [58]. The correlation occurred only with HSV1, not with CMV and HHV6 antibodies, and there was no correlation of the latter with either magnetic resonance imaging data (indicating cortical volumes) or with clinical parameters in AD patients [56,57,58]. Recently, Lovheim et al. [59] found an association between HSV carriage, as shown by the presence and level of serum IgG antibodies, and declining episodic memory function; the association was particularly strong among APOE-ε4 carriers.

Two other studies—by Lopatko Lindman et al. [60], and Linard et al. [61], examined these features in relation to the combined risk of AD conferred by HSV1 and APOE-ε4. Lopatko Lindman et al. investigated a large nested case-control study and found an increased risk of developing AD in the association of APOE-ε4 heterozygotes and anti-HSV1 IgG carriage (OR 4.55, *p* = 0.02), compared with APOE-ε3 homozygotes, but none for carriage of either factor alone, nor for anti–HSV2 IgG, nor anti–CMV IgG. APOE-ε4 homozygosity increased the risk greatly, while there was no significant association with APOE-ε2 homozygosity. In addition, a calculated genetic risk score, based on nine additional risk genes, interacted with anti–HSV1 IgG, increasing the risk of AD (OR 2.35, *p* = 0.01). Linard et al. surveyed a prospective cohort and reported that among APOE-ε4 carriers—characterised by the authors as having a high frequency of HSV1 reactivation—those positive for IgM or those who had high IgG levels, had an increased risk of AD. APOE-ε4-negative subjects showed no significant association.

A novel genetic approach was reported by Pandey et al. [62], who found that homozygous carriers of GM17, a variant of the IgG1 antibody gene, have a fourfold greater risk of AD than have non-carriers, independently of APOE-ε4 risk. GM17 has an increased affinity for a decoy receptor of HSV1, a complex of two glycoproteins, gE-gI on the surface of the virion and infected cells, that binds the Fc region of host IgG (and is implicated in cell-to-cell spread of virus). This enables HSV1 to evade antibody-mediated host responses—the cell’s immune defences. Other evidence supporting the possibility that HSV1-specific antibodies have a protective effect against AD development includes the fact that AD incidence increases with age-dependent blood-brain barrier (BBB) injuries, although detected in normal brain, are more pronounced in mild cognitive impairment (MCI) subjects [63,64], so a relatively high concentration of HSV1-specific antibody could limit viral reactivation in brain regions where the BBB is disrupted. (Montagne et al. [64] have since shown that in APOE-ε4 carriers, blood-brain-barrier breakdown occurs in the hippocampus and medial temporal lobe in cognitively unimpaired carriers but breakdown is greater in cognitively impaired subjects, and is unrelated to CSF or PET measurements of Aβ or tau pathology; they suggest that the breakdown contributes to APOE-ε4-associated cognitive decline, independently of Alzheimer’s disease pathology.) The efficacy of humoral responses is also determined by antibody avidity. The HSV1-IgG avidity index was found to be higher in subjects with MCI than in normal subjects and AD patients [65], and HSV1-specific antibody avidity was significantly higher at baseline in MCI subjects who did not develop AD than in those who did develop the disease [66].

## 10. Relationship of APOE and of Infection, and Infectious Burden to Cognition

APOE-ε4 was the first major risk factor found for AD, and a decline in cognition is a major feature of AD. Whether or how this risk and this symptom interact and whether and how infection is involved are major questions. In fact, two interesting studies on cognition and infectious agents and two on APOE and cognition have recently been published. A very intriguing one is that of Zhao et al. [67] describing their investigation of a multi-ethnic cohort, the Northern Manhattan Study (NOMAS). Their aim was to find if APOE-ε4 modifies the association between infectious burden (IB) and cognitive outcome in the population. IB was assessed by a quantitative weighted index of exposure to common pathogens associated with vascular risk, infectious burden index (IBI), and by serology for individual infections. They had previously found an association between an IBI, a composite serologic measure of exposure to common pathogens linked to stroke risk, and poor cognitive performance on global cognitive measures. Subsequently, using detailed full neuropsychological testing, they found an association between IB and the executive function domain, and also decline in memory over time. Serologies for IB were investigated using ELISA for Chlamydia pneumoniae, Helicobacter pylori, cytomegalovirus, and HSV1 and HSV2. IgG titres were used for all the pathogens except *C. pneumoniae*, for which IgA titres were used. Cognition was assessed by completion of the MMSE at baseline, and by a full neuropsychological test battery, comprising cognitive domains of memory, processing speed, language, and executive function, after a median follow-up of approximately 6 years.

Adjusted linear and logistic regressions estimated the association between IBI and cognition, with a term included for the interaction between APOE-ε4 and IBI. The data revealed interactions between IBI and APOE-ε4 (*p* = 0.07), and HSV1 and APOE-ε4 (*p* = 0.02) for processing speed in those subjects who had full neuropsychological test results. IBI was associated with slower processing speed among non–ε4 carriers, but not among APOE ε4 carriers. HSV1 seropositivity was associated with slower processing speed among non–ε4 carriers, but not among APOE ε4 carriers. Two intriguing features of the study were discussions on the relevance of differences in APOE-ε4 frequency amongst the different ethnic groups, and the relevance of the pleiotropy of APOE-ε4 (see below), with APOE-ε4 acting as a modifier of the association between IBI and cognition, and in turn being modified to some extent by ethnicity. The authors, however, seemed unaware that APOE modulates the consequences of infection in a number of infectious diseases [20], not just in the cited protective action of ε4 in HCV-infection of liver [68]. In addition, in AD patients, their suggestion that APOE-ε4 carriers might have increased susceptibility to HSV1 is unlikely to be correct, in view of the very high proportion of people infected with HSV1.

Another study on a related theme [59] was carried out to find if there is a link in early-stage AD between episodic memory decline over a number of years old and HSV and APOE-ε4, using cross-sectional and longitudinal studies on a large cohort. Episodic memory impairment is one of the first preclinical signs of AD, and is strongly associated with increased age-related AD risk, although its course varies greatly among individuals. Cross-sectional analyses showed an age-dependent association of HSV presence (IgG seropositivity) with lower episodic memory function (using a composite measure of 5 episodic memory tasks), particularly among APOE-ε4 carriers (*p* = 0.008). Longitudinal analyses revealed an increased risk of episodic memory decline in older HSV carriers (*p* < 0.001). The results thus support an association between HSV carriage and declining episodic memory function, especially among APOE 4 carriers.

The study of Gharbi-Meliani et al. [69] aimed to find whether the association between APOE-ε4 zygosity and cognition function is modified between midlife and old age. This allele is the strongest genetic risk factor for late onset Alzheimer’s disease, the prevalence of ε4 allele heterozygotes in the populations in the West being about 25–30%, and of ε4 homozygotes about 2%, so there are many people at appreciable risk of the disease. However, apart from the link with dementia, the association between APOE ε4 and change in cognition over the adult lifespan is uncertain. The survey investigated 5561 subjects in the Whitehall II Study, an ongoing cohort study of persons originally employed by the British Civil Service, all Caucasian. The authors commented that some previous studies had shown accelerated cognitive decline in APOE-ε4 homozygotes but not heterozygotes. In addition, the association between APOE ε4 and cognition has been thought to depend on age, though the relationship is unclear, some but not all studies reporting better cognition performance at young ages among ε4 carriers. The authors stated that the age-dependent association of APOE ε4 with cognitive performance could be explained by the antagonistic pleiotropy hypothesis, which postulates that the effect of a gene on health depends on the stage of life. However, as they said, explanations or deductions are limited, because most research on APOE had been made on subjects of age 65 years or older, who have been followed for less than 10 years.

The authors concluded that their longitudinal study produced two main findings. Firstly, it confirmed that the ε4 allele of APOE is associated with accelerated cognitive decline over the adult life course in both heterozygotes and homozygotes, irrespective of occurrence of dementia. The cognitive performance of e4 carriers was noticeably worse than that of non-ε4 carriers, from 65 years of age for homozygotes and from 75 years for heterozygotes. Secondly, a seemingly paradoxical effect of APOE ε4 was detected in heterozygotes, who performed better on the global cognitive score than non-ε4 carriers up to the age of 55 years. More detailed analyses suggested that the younger ε4 heterozygotes performed better in tests that involved executive function (reasoning, phonemic fluency). These results provide support for the antagonistic pleiotropic hypothesis. The authors suggest that APOE-ε4 carriage in those younger than about 55 might confer an advantage, especially in reasoning and psychomotor speed, which might have helped to conserve the ε4 allele during pre-historic times, when life expectancy was below 50 years. They too were probably unaware, like Zhao et al. [67], that APOE has been shown to modulate response to several infectious diseases [20] and in the case of HCV-induced damage of liver, that APOE-ε4 confers protection, so that its carriage might have an evolutionary advantage against certain other pathogens.

There is yet another report that seems not to consider the relevance of APOE-ε4 carriage in respect to HSV1 and AD. Murphy et al. [70] investigated the relationship between HSV1 and risk of cognitive decline, dementia and AD using data from two of the four cohort of the Rotterdam study. The authors measured serum IgG antibodies to HSV1 collected between 2002 and 2005, and changes in cognitive performance during 2 consecutive examinations 6.5 years apart. The association of HSV1 with risk of dementia and AD in the 1915 non-demented subjects (mean age 71.3 years), was followed on average for 9.1 years during which, 244 participants developed dementia, 203 of whom were diagnosed with AD. Controls were seronegative people. HSV1 seropositivity was associated with decline in global cognition, as well as in separate cognitive domains. However, HSV1 seropositivity was found not to be associated with risk of dementia (adjusted HR 1.18, 95% CI 0.83–1.68), nor with risk of AD. They therefore suggested that HSV1 is associated only with subtle cognitive disturbances but not with greater cognitive disorders that result in dementia. Their discussion indicates though that they were unaware that the relationship between HSV1 and AD depends on carriage of an APOE-ε4 allele and on HSV1 presence in brain. As stressed at the start of the present article, HSV1 alone confers little or no risk of AD. That fact is obvious when one considers that the virus infects about 80% of the Western world’s populations by the age of 60; if all those infected were as a consequence to develop AD, 80% of these populations would succumb to the disease: a terrible thought. Further, their comparison with the Taiwanese study of Tzeng et al. (2018) was not valid, since the latter compared HSV1-infected carriers of (almost certainly) APOE-ε4 with people uninfected with the virus

## 11. Relationship of HSV1-Induced Oxidative Stress, Autophagy, and Apoptosis to AD

Major disruptions to cellular processes that are thought to be involved in AD and their possible causes include oxidative stress (OS), which has been strongly implicated in AD pathology, and HSV1, which is known to cause such stress. AD brains have high levels of polyunsaturated fatty acids, the substrate for lipid peroxidation, also a high rate of oxygen usage, low level of antioxidants and they show increased levels of oxidative stress markers, such as various protein derivatives, and lipid oxidation products. Herpes simplex encephalitis (the rare but very serious acute HSV1 infection of brain) increases production of reactive oxygen and nitro species, which contributes to oxidative stress also, HSV1-infected neural cell cultures produce increased levels of reactive oxygen species (ROS), resulting in neuronal oxidative damage including lipid peroxidation. Recently, Protto et al. [71] investigated oxidative damage in the mouse model set up by the same group [72], in which multiple HSV1 reactivations were achieved by thermal stress. Protto et al. used biochemical and redox proteomic approaches. They found that the reactivations cause oxidative modification in lipids and proteins in the cortex, most of the proteins being involved in cellular processes such as energy metabolism, protein folding, degradation process, and cell structure—pathways linked to AD onset and progression. They concluded that repeated reactivation in brain might contribute to neurodegeneration through oxidative damage such as accumulation of misfolded proteins, perturbation of the cytoskeleton network and of clearance mechanisms for oxidised proteins and DNA, leading to cell translation machinery being used for synthesising viral instead of cell proteins.

The likelihood that HSV1 effects on clearance (of damaged proteins) and on autophagy might relate to AD was proposed in 2008 [73]. It was postulated that the virus not only generates Aβ and P-tau in cells but also, via the HSV1 protein ICP34.5, disrupts autophagy, preventing their degradation, thereby leading to their accumulation and deposition. Subsequently, details of the impairment of clearance mechanisms of the lysosome system by HSV1 via oxidative stress have been reported in many studies, most recently by Kristen et al. [74]. They found in a cell model of AD that HSV1 and OS increased lysosome load, reduced activity of lysosomal hydrolases, affected cathepsin maturation, and inhibited endocytosis-mediated degradation of the epidermal growth factor receptor, indicating impairment of the lysosome system. They drew a similar conclusion from functional genomic analysis.

Duarte et al. [75] described the relationship of HSV1 infection to apoptosis. They commented that although apoptosis is considered to be a result of neurodegeneration, alterations in signalling pathways related to apoptosis have been widely implicated in neurodegenerative diseases such as AD; they could be caused by HSV1 modulation of neuronal apoptosis during infection: host cells use apoptosis during viral infection in order to eliminate the virus and to combat this, HSV1 modulates apoptosis-related pathways at multiple stages after infection of neurons, during both acute and latent infection, leading to either the induction or the blockage of apoptosis. The virus can thus manipulate neuronal survival and its own persistence. Inhibition of apoptosis can occur via the HSV1 protein ICP34.5, which dephosphorylates eIF2α, thereby blocking the shutdown of host cell and of viral protein synthesis and preventing apoptosis, while enhancing autophagy; it can occur also via several other viral proteins, and the HSV1 latency-associated transcript, LAT. However, Doll et al. [76] have shown that in HSV1-infected mouse trigeminal ganglia neurons, infectious virus is eliminated after reactivation, through destruction of neurons containing HSV proteins, by phagocytic macrophages and microglia.

The involvement of mitochondrial damage in AD was discussed by Reddy et al. [77]. They proposed that mitochondrial energy metabolism is impaired by the expression of mutant APP and/or Aβ, and that consequent up-regulation of mitochondrial genes and of apoptotic-associated genes is a compensatory response and an early change in AD. Recently, Pradeepkiran and Reddy [78] described impairment of mitophagy, a process whereby damaged mitochondria are selectively removed from cells, so that progressive accumulation of defective organelles and damaged mitochondria occurs. In AD, increased levels of Aβ and P-tau can induce production of reactive oxygen species, ROS, causing excessive fragmentation of mitochondria and promoting defective mitophagy. As to HSV1 and mitophagy, only one study has investigated effects of the virus on mitophagy: Waisner and Kalamvoki [79] found that HSV1 evades the host mechanisms of autophagy and mitophagy, which are defence mechanisms against pathogens, through the downregulation of the autophagy adaptor protein, sequestosome, and of the mitophagy adaptor, optineurin, via the HSV1 protein, ICP0.

## 12. Conclusions

The evidence for HSV1 in brain of APOE-ε4 carriers conferring a strong risk of AD continues to grow, with no experimental counter-evidence published in very recent years—as far as the present author is aware. Details of the precise mechanisms whereby HSV1 operates over the years to cause the development of the disease are yet to be discovered: a challenge bearing in mind the bewildering array of effects of the virus and the very unfortunate inability at present to detect it even when reactivated in the brain in life. Elucidation of the effects of HSV1 during latency, and especially development of an adequate model system for latency would be invaluable, as too would be finding what effects, if any, HSV1 alone has in the absence of apoE-ε4. Nonetheless, all the evidence provides increasing support for further clinical trials which, in this author’s opinion, should treat patients (at least HSV1-seropositive APOE-ε4 carriers) not only with VCV, but also with fucoidan, in view of its very different mode of action from that of blockers of HSV1 DNA replication. An alternative to VCV would be usage of a helicase primase inhibitor: interestingly, a study of a new such inhibitor, IM-250, which provides in animals sufficient brain exposure and a potentially superior dose regimen of once daily or even once weekly (rather than the usual twice daily) is in press [80]. Equally, or even more exciting would be a trial to investigate the apparent protective effects of vaccination, particularly of BCG, against the development of dementia, and a trial treating symptomatic sufferers of HSV1 with antiviral agents in their middle years, well before any signs of dementia are detectable. Prevention of dementia would a truly marvellous outcome.
